# Potential of Caffeine in Alzheimer’s Disease—A Review of Experimental Studies

**DOI:** 10.3390/nu13020537

**Published:** 2021-02-06

**Authors:** Piotr Londzin, Milena Zamora, Beata Kąkol, Aleksandra Taborek, Joanna Folwarczna

**Affiliations:** Department of Pharmacology, Faculty of Pharmaceutical Sciences in Sosnowiec, Medical University of Silesia, Katowice, Jagiellońska 4, 41-200 Sosnowiec, Poland; milena.zamora95@gmail.com (M.Z.); beata744@interia.eu (B.K.); taborek.aleksandra@gmail.com (A.T.); jfolwarczna@sum.edu.pl (J.F.)

**Keywords:** Alzheimer’s disease, experimental Alzheimer’s disease models, caffeine, coffee

## Abstract

Alzheimer’s disease (AD) is the most common type of dementia leading to progressive memory loss and cognitive impairment. Considering that pharmacological treatment options for AD are few and not satisfactory, increasing attention is being paid to dietary components that may affect the development of the disease. Such a dietary component may be caffeine contained in coffee, tea or energy drinks. Although epidemiological data suggest that caffeine intake may counteract the development of cognitive impairment, results of those studies are not conclusive. The aim of the present study is to review the existing experimental studies on the efficacy of caffeine against AD and AD-related cognitive impairment, focusing on the proposed protective mechanisms of action. In conclusion, the reports of studies on experimental AD models generally supported the notion that caffeine may exert some beneficial effects in AD. However, further studies are necessary to elucidate the role of caffeine in the effects of its sources on cognition and possibly AD risk.

## 1. Introduction

Alzheimer’s disease (AD) is the most common type of dementia, accounting for 50–70% of neurodegenerative dementia cases. AD leads to a progressive loss of memory and cognitive abilities [1].

It is believed, especially taking into account very limited therapeutic options, that dietary interventions or nutraceuticals may be promising in the prophylaxis and treatment of cognitive impairment of ageing, including AD [2,3,4,5,6,7]. The effects of dietary components are usually evaluated based on dietary questionnaires, and the results of those studies, as well as the results of their meta-analyses are often not conclusive. The number of randomized controlled trials concerning phytochemicals is low [4]. On the other hand, numerous experimental studies indicated phytochemicals as potentially useful in the prophylaxis of AD, including flavonoids, phenolic acids, carotenoids, curcumin, resveratrol, and some alkaloids (for comprehensive review—see [4,6]). From among those phytochemicals, the effects of caffeine seem to be the most thoroughly examined and best documented.

Caffeine (1,3,7-trimethylxanthine) is a purine alkaloid, commonly consumed on a daily basis. Caffeine is probably the most commonly used psychoactive substance/psychostimulant [8,9,10]. It is found in coffee (*Coffea* L.) beans, cola (*Cola acuminata* (P. Beauv.) Schott and Endl.) nuts, tea (*Camellia sinensis* (L.) Kuntze) leaves and yerba mate (*Ilex paraguariensis* A.St.-Hil.) leaves, as well as guarana (*Paullinia cupana* Kunth) seeds and cocoa (*Theobroma cacao* L.) beans [4,10]. The main dietary source of caffeine is coffee, tea and yerba mate [11]. Caffeine is also present in soft drinks (cola-type) and energy drinks, used mainly by younger individuals [8]. The mean caffeine intake varies between countries, however it has remained stable in adults in the last 10–15 years [12]. According to the recommendations of the European Food Safety Authority (EFSA), the daily caffeine intake up to 400 mg is considered safe for healthy adults [13,14].

Numerous health-promoting activities are attributed to coffee and caffeine. A recent umbrella review of meta-analyses indicated, among others, beneficial role of coffee in reducing risk of type 2 diabetes mellitus (T2D), cardiovascular diseases, some cancers and Parkinson’s disease [15,16]. The results of epidemiological and experimental studies suggest also a possible beneficial effect of caffeine in the prevention of AD [17,18,19,20]. However, human studies concerned caffeine contained in the diet and cannot isolate caffeine’s effects from countless lifestyle choices people make.

Here we would like to focus on the results of experimental studies of caffeine, carried out in order to evaluate its potential in the prophylaxis and treatment of AD. A literature search was conducted on the PubMed electronic database. Articles presented in languages other than English were excluded. Results of all studies on caffeine effects in different AD experimental models, published until December 2020, and found in the PubMed electronic database, were referred to in the present review.

## 2. Caffeine—Main Mechanisms of Action

The mechanism of action of caffeine is complex. Caffeine is a non-selective antagonist of adenosine receptors (mainly A_1_ and A_2A_), demonstrating a structural similarity to adenosine. The blocking of these receptors modulates glutamatergic, cholinergic, dopaminergic, serotoninergic and noradrenergic neurotransmission [8,21,22]. The blockade of adenosine receptors is observed in lower concentrations of caffeine (<250 µM) [22]. Moreover, caffeine is an agonist of ryanodine receptors (RyRs), stimulation of which increases the release of Ca^2+^ from the endoplasmic reticulum [23,24]. Caffeine is also a non-selective competitive inhibitor of phosphodiesterases (PDEs), the enzymes degrading cyclic adenosine monophosphate (cAMP), which leads to increases in the cell cAMP concentration [8,23]. However, the effect of caffeine through the signaling pathways associated with the stimulation of RyRs and the blocking of PDEs is only possible at higher doses (plasma concentration >250 µM) [22,23,25]. Caffeine also interferes with γ-aminobutyric acid type A (GABA_A_) receptors [24,26,27]. Caffeine protects against cell damage, exerts antioxidant effects, reducing oxidative stress markers [23,28]. Caffeine may exert also anti-inflammatory activity, decreasing proinflammatory (C-reactive protein, interleukin (IL)-1β, IL-6, IL-18, tumor necrosis factor α—TNF-α) and increasing anti-inflammatory (IL-10, adiponectin) marker levels [23,29,30].

## 3. Alzheimer’s Disease (AD)

AD is characterized by personality disorders, amnesia, dementia and cognitive impairment [31]. Brain atrophy observed in AD results from the synaptic degeneration and neuronal cell death. Dysfunctions occur mainly in the brain regions that play a major role in memory and spatial orientation, i.e., in the hippocampus, striatum, cerebral cortex, thalamus and amygdala [32,33]. Besides cognitive dysfunctions, the majority of patients with AD suffer from behavioral and psychological symptoms of dementia (BPSD). BPSD include depression, apathy, anxiety, sleep changes, hallucinations, delusions and agitation [34,35,36].

There are two major forms of AD—familial (or early-onset) and sporadic. Familial AD (FAD), 5% of AD cases [35,37], is caused by mutations in genes encoding three proteins—amyloid precursor protein (APP), presenilin 1 (PS1) and presenilin 2 (PS2) [38]. The cause of the sporadic forms of AD is unknown; the majority of them develop after 65 years of age [39]. The ε-4 allele of the apolipoprotein E (APOE) is a risk factor in the pathogenesis of late-onset of AD, playing an important role in amyloid β (Aβ) brain metabolism (APOE ε-4 exacerbates deposition of Aβ in the brain and tau-mediated neurodegeneration) [40,41,42]. Among various risk factors associated with AD, T2D, traumatic brain injury, cerebrovascular disease, hypertension, dyslipidemia, obesity and metabolic syndrome have been demonstrated [43,44]. There is a hypothesis that the sporadic type of AD is triggered by dysfunctional insulin signaling in the brain; it has been proposed that sporadic AD may be considered the brain type of diabetes [45] or type 3 diabetes [46,47].

The most popular hypothesis on the pathogenesis of AD is the amyloid hypothesis, which assumes an important role of Aβ deposits in the form of senile plaques [48,49]. In fact, the neuropathological hallmarks of AD are the presence of extracellular neuritic plaques, consisting of deposits of Aβ peptides and intraneuronal neurofibrillary tangles (NFTs) composed of aggregated and often truncated, and hyperphosphorylated tau protein [1,50,51].

Aβ is formed as a result of incorrect fragmentation of the amyloid precursor protein (APP) processed by β-secretases (β-site amyloid-precursor-protein-cleaving enzyme-1—BACE-1 and BACE-2) and γ-secretases [52]. Senile plaques activate microglial cells and induce inflammation [49,52,53]. In AD, the tau protein uncouples from microtubules, aggregates into tangles and inhibits microtubular transport [50]. Hyperphosphorylation of tau protein causes dysregulation of the neuronal system and axon damage [52]. Also, neuroinflammation is considered to be involved in the pathogenesis of AD [53]. Proinflammatory cytokines (TNF-α, IL-1 and IL-6), reactive oxygen species (ROS) and NO production leads to dysfunction of the blood–brain barrier (BBB) and the influx of immunocompetent cells [32]. The associated oxidative stress causes lipid and neuronal protein oxidation [48,52]. Inflammation-related biomarkers are present in the cerebrospinal fluid, peripheral blood and brain in AD patients [49,53,54].

Neurodegenerative disorders in AD lead to decreases in the levels of neurotransmitters. An important role in the pathogenesis of AD is a reduction of acetylcholine (ACh) content. The dysfunction of cholinergic system is related to degeneration of the nucleus basalis of Meynert cholinergic neurons caused by NFTs formation. The presence of NFTs and Aβ plaques leads to cholinergic synapse loss. Moreover, despite the preservation of postsynaptic M_1_ muscarinic receptors (the level of presynaptic M_2_ receptors is decreased) in the cerebral cortex, their functions are impaired. Also, progressive decrease in the number of nicotinic receptors in the cerebral cortex is observed. Deficits in cholinergic neurotransmission contribute to the learning, memory and attention impairment [55,56]. Moreover, it has been demonstrated that interaction between cholinergic and glutamatergic transmission is involved in Ca^2+^-dependent neuroprotection [56]. The glutamatergic system is controlled by various mediators, including adenosine and ACh [56,57,58]. In AD, there is a decrease in the number of the N-methyl-D-aspartate (NMDA) receptors resulting in an imbalance between synaptic and extrasynaptic NMDA receptors [59,60]. Malfunctioning glutamatergic neurons release too much glutamate, which over-stimulates the extrasynaptic NMDA receptors leading to the neuronal cell death and synaptic loss [59]. Moreover, cholinergic neuronal loss leads to dopaminergic transmission dysfunction which is correlated with psychiatric symptoms of AD (BPSD) [55,56]. Also, the loss of noradrenergic neurons in the locus coeruleus caused by the tau protein accumulation results in the progression of AD. Noradrenergic receptors (mainly α_2_ receptor activation or β receptor blockade) are involved in the Aβ-related neuropathology. Upregulation of adenosine receptors (A_1_R and A_2A_R) is also observed in AD. Activation of adenosine receptors affects synaptic neurotransmission and various neurotransmitters release (ACh, glutamate) [58]. Moreover, it disrupts learning and memory processes and leads to cognitive disorders [58,61]. Also, dysregulated signaling by other purinergic receptors may be involved in the pathomechanism of AD. Activation of P2X7 receptors by high levels of adenosine triphosphate—ATP or its metabolites (Aβ accumulation induces increased release of ATP) leads to neuroinflammation, i.e., increased level of IL-1, IL-6, IL-18, TNF-α and interferon-γ, and neurodegeneration [62].

Summing up, cholinergic and glutamatergic transmission disorders are associated with impaired cognitive processes, while disorders of dopaminergic, serotonergic and noradrenergic transmission are responsible for the symptoms associated with dementia, such as depression, apathy, anxiety and psychotic symptoms [63].

Epidemiological studies demonstrated higher occurrence of AD (2:1 women to men ratio) and risk of developing AD in women compared to men. It is probably related to the longer life expectancy for women. In addition, faster cognitive decline and longer life span after diagnosis of mild cognitive impairment or AD dementia is observed in elderly women in comparison to men [64,65,66]. More aggressive behaviors and higher mortality is demonstrated in men, whereas women suffer from more severe symptoms of affective disorders [67].

The treatment options of AD are very limited. Currently, only four drugs are approved by Food and Drug Administration (FDA) and European Medicines Agency (EMA) for AD treatment. These include three drugs from the group of acetylcholinesterase (AChE) inhibitors (donepezil, rivastigmine and galantamine) and memantine, a selective and non-competitive NMDA receptor antagonist [68,69]. AChE inhibitors increase ACh levels in the synaptic cleft and partially improve cognitive function and the quality of a patient’s life [55]. Memantine restores NMDA receptor function and exerts neuroprotective effects, reducing the intracellular influx of calcium ions [70,71]. There is a great need to search for new therapeutic and prophylactic approaches to AD.

Taking into account the postulated pathomechanism of AD, it is believed that antioxidant nutraceuticals may have beneficial effects in the prevention of AD. The best-known antioxidants include plant polyphenols such as curcumin, flavonoids and phenolic acids. Experimental studies and/or population studies suggest the possibility of beneficial effects of curcumin [3,72,73,74], ferulic acid [3,75,76,77], epigallocatechin 3-gallate (EGCG) [3,78,79,80] and caffeine [18,19,20,21,23,81,82,83] in AD treatment and prevention.

## 4. Caffeine in Alzheimer’s Disease

The studies on the effects of caffeine consumption (coffee, tea, other sources) on the cognitive impairment, mild cognitive impairment, dementia and AD led to differential conclusions. Although the review articles and meta-analyses indicated rather favorable effects of caffeine-containing dietary sources on those disorders, their conclusions were not unequivocal [84,85,86,87,88,89,90]. For example, a latest systematic review supported the notion that green tea intake might reduce the risk of those disorders [86], but the meta-analysis of observational studies did not demonstrate the effect of tea drinking on AD specifically [87]. Similarly, the meta-analyses concerning coffee intake led to differential conclusions. A meta-analysis of prospective cohort studies demonstrated a “J-shaped” association between coffee intake and the occurrence of cognitive disorders (the lowest risk with daily consumption level of 1–2 cups of coffee) [88]. On the other hand, another analysis of prospective cohort studies found that higher coffee consumption was associated with reduced risk for AD [89]. The most recent meta-analysis of prospective studies did not support an association between coffee consumption and a risk of AD and overall dementia [90]. Moreover, it seems worth mentioning that studies using Mendelian randomization, which is a genetic epidemiological method, did not provide the evidence for a causal effect of habitual coffee consumption on global cognition or memory [91]. In fact, surprisingly, one study even suggested an association between genetically predicted higher consumption of coffee and higher risk of AD (the report concerned analysis of genome-wide association studies of the potentially modifiable risk factors of AD) [92].

It should be emphasized that other than caffeine active constituents of coffee or tea (like phenolic acids, EGCG, trigonelline) may contribute to their effects on cognition/AD risk [4,93]. Decaffeinated coffee constituents have been demonstrated to exert potential beneficial effects in neurodegenerative diseases [94]. However, decaffeinated coffee was not associated with a protective effect on cognitive performance in older people [95]. The data from the human studies do not allow to conclude on the role of caffeine itself in the modulation of AD risk. However, there is growing evidence on the effects of caffeine on cognition and AD development coming from numerous experimental studies.

## 5. Experimental Models of AD

It is crucial to use experimental models faithfully mimicking the pathologies of investigated diseases. In case of AD, it is problematic. None of the experimental AD animal models fully reflects complete disorders and cognitive impairments characteristic for human AD. Although FAD accounts for about 5% of all disease cases only, the majority of potential therapies has been investigated in transgenic mouse models of AD [42].

Transgenic technologies allowed mouse and rat models to be created, based on the mutations of APP, presenilin (PS), APOE and tau protein genes [37,42,96]. Mouse models are the most commonly used in the experimental studies on AD, due to their low prices, relatively short life span and similar AD symptoms to the human disease [96]. Histopathological changes in the brain tissue, depending on the mutation used, are characterized by the presence of Aβ plaques, tau protein deposits and hippocampus atrophy [1,56,97].

APP mutations causing cognitive deficits in mice and rats are related to an increased Aβ production, increased Aβ accumulation (in the blood or cerebral vessels, in neurons of the cortex and hippocampus), promotion of fibrillogenic and toxic Aβ_1-42_ form or modification of the Aβ_1-42_/Aβ_1-40_ ratio, depending on the type of mutation [96,98,99,100]. Pathogenic PS1 or PS2 mutations increase the levels of Aβ_1-42_ form in vitro, but do not promote Aβ plaques aggregation in vivo in mice. Several double-transgenic (2xTg) mouse models have been developed by crossing APP and PS1 transgenic mice, demonstrating accelerated Aβ accumulation leading to the impairment of cognitive functions [98,100,101].

In the human tau protein (h-tau) transgenic model, the mice tau gene is replaced by the human gene correlating with NFTs development and neurodegeneration [37,42,100,102,103]. The most commonly used tau transgenic model is THY-Tau22 model in which progressive development of the hippocampal tau pathology is observed. Hyperphosphorylated hippocampal tau protein and neuroinflammation results in an age-dependent memory impairment [103,104,105].

A triple-transgenic mice model of AD (3xTg-AD) is a combination of three mutations resulting in the age-related progressive neuropathy including accumulation of Aβ plaques and NFTs, resulting in the cognitive deficits [98,106,107], whereas the 5xFAD transgenic model concerns five mutations exhibiting severe pathology with accelerated Aβ accumulation, senile plaques formation and neuronal loss [37,98,108].

The number of rat genetic models is much lower (for example TgF344-AD, single Tg UKUR28, double Tg UKUR25). In the TgF344-AD rat model (human APPswe and human PS1ΔE9 mutations) accumulation of Aβ plaques, presence of NFTs, gliosis, increased tau protein level and decreased basal hippocampal synaptic transmission was observed [109,110]. Aβ accumulation in the hippocampus and cortex, and increased levels of phosphorylated mitogen-activated protein kinase/extracellular signal-regulated kinase 2 (MAPK/ERK2) were observed in UKUR28 (human APPswe mutation) and UKUR25 (human APPswe and human PS1 Finn mutations) transgenic rat models [97,99,111].

Moreover, several non-mammalian species, including nematode (*Caenorhabditis elegans*), zebrafish (*Danio rerio*) and fruit fly (*Drosophila melanogaster*) and have been used to create transgenic models of AD [112].

In spite of a large number transgenic animal models of AD, it should be pointed out that results of the preclinical studies on those models have been rather not satisfactorily translated into clinical outcomes [37].

Several pharmacological models of sporadic AD have been used. Those models included: administration of aluminum chloride per os (*p.o.*) [113], intraperitoneal (*i.p.*) administration of scopolamine [114,115], intracerebroventricular (*i.c.v.*) injections or infusions of Aβ [116,117], *i.c.v.* injections of streptozotocin (STZ) [47,118], subcutaneous (*s.c.*) injections of *d*-galactose [78,119], *i.p.* injections of MK-801 (a non-competitive NMDA receptor antagonist) [120], olfactory bulbectomy (OBE) [121] and *i.c.v.* injections of lipopolysaccharide (LPS) [116,122,123,124] or colchicine [116]. A model of senescence-accelerated rodents (OXYS rats) is also used as a model of sporadic AD [125,126]. It is a non-transgenic model established by inbreeding of highly susceptible rats with spontaneously developing cataract and accelerated senescence syndrome. OXYS rats demonstrate learning and memory deficits, decreased locomotor activity and progressive cognitive impairment [121,125,126].

To summarize, it should be pointed out that sophisticated transgenic experimental models of AD are used in preclinical in vitro and in vivo studies on novel drugs or compounds potentially useful in the prevention or treatment of AD. However, those models reflect only genetic changes present in familial AD, whereas the sporadic form of AD is much more common. On the other hand, mostly rather simplistic models of sporadic AD are currently being used in the experimental studies. Some of them are controversial, for example the scopolamine model [112,127]. It seems that there is a need for development of new, reliable models of sporadic AD.

### 5.1. Effects of Caffeine in Experimental Animal Models of AD

#### 5.1.1. Transgenic Rodent Models

The effects of caffeine have been studied in various transgenic models of AD, including APPswe, double transgenic (2xTg) APPswe/PS1, THY-Tau22 and 3xTg models (Table 1) [104,105,128,129,130,131,132,133,134,135,136].

The most commonly used transgenic models of AD in animals is the APPswe mouse model. In the APPswe model, mice develop cognitive deficits, learning impairment and age-dependent spatial memory dysfunction [37,98,129,130,131]. Expression of human APP in transgenic mice model enable Aβ plaques deposition and synaptic and neuritic dystrophy [130,131,137].

In studies on the APPswe transgenic mice model, caffeine intake prevented cognitive impairment, memory deficits and exerted neuroprotective activity [128,129,130,131,133]. The neuroprotective caffeine activity associated with reduced Aβ production resulted from the inhibition of BACE-1 and γ-secretase [129], suppressed neuronal cell death, reduced caspase-3 activity [131], activation of cAMP/PKA (cyclic adenosine monophosphate/phosphokinase A) signaling pathway and stimulation of cAMP response element-binding protein (CREB) phosphorylation in the striatum [128]. Two-week caffeine treatment in APPswe mice exerted antiapoptotic activity (decreased the expression of phosphorylated c-Jun N-terminal kinase (JNK) and phosphorylated extracellular signal-regulated kinase (ERK) in the striatum and frontal cortex) [128]. Long-term treatment with caffeine improved mitochondrial functions in the hippocampus, cerebral cortex and striatum in APPswe transgenic mice [133]. No effect on A_1_R and A_2A_R hippocampal density was observed in APPswe transgenic mice treated with caffeine [129]. Caffeine treatment in N2a neuroblastoma cells transfected with mutant APPswe indicated slight antioxidant activity associated with reduced ROS production [133].

Chronic intake of crude (containing 95.95% caffeine) and pure caffeine prevented memory disorders in APPswe mice model. However, only crude caffeine reduced the level of Aβ and formation of Aβ plaques in the hippocampus. Crude caffeine increased the level of ATP (an indicator of cell survival), reduced caspase-3 activity and reduced Aβ-induced neuronal cell death [131]. Crude and pure caffeine increased the number of cholinergic neurons in Aβ-treated cultures [131]. Greater effects of crude caffeine may result from the activity of other than caffeine components. Phenolic substances (about 1%) may be responsible for antioxidant activity of crude caffeine which exhibited much higher values than pure caffeine in the oxygen radical absorbance capacity (ORAC) assay [131,138]. Moreover crude caffeine strongly inhibited cyclooxygenase-2 (COX-2), whereas pure caffeine did not inhibit the enzyme [138]. The authors concluded that substantial antioxidant and anti-inflammatory effects of crude caffeine are crucial in preventing cognitive and memory impairment in APPswe transgenic mice [131].

Studies on caffeine were also performed on 2xTg and 3xTg animal models. Double transgenic mice indicate rapid rate and early onset of AD associated with cognitive impairment [98]. For example, the double transgenic APPswe/PS1 mice model is related to Aβ plaque formation in the cortex and hippocampus [37,98]. In those mice spatial learning and memory assessed in the water maze test worsened [132].

Acute intake of caffeine by APPswe/PS1 transgenic mice resulted in a reduced Aβ production (by inhibiting γ-secretase and BACE-1) and reduced Aβ_1-40_ and Aβ_1-42_ levels in plasma (the same effects were observed in transgenic APPswe mice). Similarly, long-term caffeine administration to APPswe/PS1 transgenic mice was related to a permanent and sustained decrease in the Aβ_1-40_ and Aβ_1-42_ levels in plasma and reduced Aβ accumulation in the brain. Chronic caffeine administration enhanced cognitive performance in the transgenic mice [134].

Acute caffeine use (administered as a coffee constituent) increased the levels of granulocyte-colony stimulating factor—G-CSF, IL-6 and IL-10 in plasma in APPswe/PS1 double transgenic mice and in non-transgenic mice. Higher levels of plasma caffeine were related to lower levels of plasma Aβ in APPswe/PS1 transgenic mice. Chronic intake of concentrated coffee improved cognitive performance and increased the level of G-CSF in plasma in APPswe transgenic mice. The authors concluded that coffee (as a source of caffeine) may protect against AD and that elevated G-CSF levels may contribute to favorable effects of coffee [136].

Also chronic treatment with caffeine in double transgenic APPswe/PS1 mice reversed memory impairment [132]. Caffeine improved spatial learning and memory, which was demonstrated by a decrease of escape latency time and longer time spent in the target quadrant assessed in the water maze test. The effects of caffeine were dose dependent. The mechanism of changes could involve the effect on the brain-derived neurotrophic factor (BDNF)—tropomyosin-related kinase receptor B (TrkB) signaling pathway, taking part in the learning and memory processes, since caffeine exerted dose-dependent increase in the expression of hippocampal BDNF and TrkB [132].

In a transgenic model of tau disorders, THY-Tau22 mice, progressively developing memory dysfunction is associated with hippocampal neuroinflammation that promotes tau protein hyperphosphorylation and aggregation [104,105,139]. Chronic administration of caffeine to THY-Tau22 transgenic mice prevented behavioral disorders and spatial memory deficits. The beneficial activity of caffeine on memory was associated with reduced hyperphosphorylated tau protein level in the hippocampus [104]. The deletion of A_2A_R improved memory, prevented spatial memory deficits and hippocampal long-term depression in THY-Tau22 transgenic mice [139]. It did not affect the tau protein level and the number of proteolytic tau fragments, which were reduced by caffeine intake in the previous study [104]. It may indicate that mechanism of caffeine in the regulation of tau-related pathology was more complex than only blockade of A_2A_R in THY-Tau22 transgenic mice [139].

Moreover, caffeine exerted anti-inflammatory activity (reduced level of proinflammatory cytokines: chemokine ligand factor 4—CCl4, chemokine ligand factor 5—CCl5 and TNF-α) and antioxidant activity (decreased activity of manganese superoxide dismutase—MnSOD and excitatory amino acid transporter 3—EAAT3 involved in the glutathione synthesis) in THY-Tau22 transgenic mice [104]. However, when caffeine was administered to pregnant THY-Tau22 transgenic rats, accelerated physiological and cognitive disorders in offspring were observed. Those results indicate that exposure to caffeine during pregnancy in rats may be a risk factor for early stages of AD [105].

Effects of caffeine were also studied in 3xTg-AD mice (carrying APPswe, PS1/M146V and tau P301L transgenes). The effects of long-term intake of caffeine in 3xTg-AD mice were assessed in multiple behavioral tests (lasting 21 days) to evaluate sensorimotor functions, exploratory activity, bizarre movements, emotional and anxiety-like behaviors, risk assessment, visual perceptual learning and reference spatial learning and memory in comparison with normal non-transgenic mice. Some of the tests were interpreted as tests concerning BPSD-like behaviors [135]. The effects of caffeine treatment in the two models were differential. Caffeine in control non-transgenic mice improved some behavioral parameters connected with cognition, exploratory and locomotor activity. Caffeine did not counteract any of behavioral disorders observed in 3xTg-AD mice, moreover some of the behavioral parameters even worsened (mostly those related to the anxiety-like behaviors). Caffeine increased horizontal locomotor activity in the open field test in non-transgenic mice and reduced it in 3xTg-AD mice; increased emotionality in the open field test, elevated plus maze and hole-board test in non-transgenic mice and reduced these parameters in 3xTg-AD mice; increased total horizontal activity in the open field test in non-transgenic mice and reduced it in 3xTg-AD mice. Caffeine increased overall spontaneous motor activity (to a greater extent at night) in circadian motor activity test only in 3xTg-AD mice [135]. The authors interpreted their results that anxiogenic effect (associated with anxiety and neophobia) induced by caffeine interfered with beneficial effects of caffeine consumption on cognition (improved visual perceptive learning, spatial learning and memory) in 3xTg-AD mice [135].

#### 5.1.2. Non-Transgenic Rodent Models

Several non-transgenic models of AD have been used to evaluate the effects of caffeine (Table 2). In a model of AD induced by *i.c.v.* administration of Aβ_25-35_, in which cognitive dysfunction is observed (Y-maze test and inhibitory avoidance task), acute caffeine administration at a higher *i.p.* dose or combined 12-day administration in drinking water, followed by acute administration at a lower *i.p.* dose, prevented Aβ-induced disorders in mice. There was no beneficial effect of the 12-day treatment with caffeine alone [117]. It was concluded that mechanism of caffeine action resulted from the blockade of A_2A_R rather than A_1_R, because similar effects were observed after a selective A_2A_R antagonist—SCH58261 administration [117]. In other studies, selective A_2A_R antagonists also prevented Aβ-induced progressive cognitive impairment and synaptic deterioration in rats [120,140].

Long-term administration of *d-*galactose to rats is associated with increased oxidative stress resulting in memory impairment, neuroinflammation and neurodegeneration. Concurrent caffeine intake improved memory functions and attenuated cognitive decline in the *d*-galactose-treated aging rats as demonstrated in the Y-maze behavioral test. Caffeine administration exerted anti-inflammatory activity (reduced level of COX-2, nitric oxide synthase-2 (NOS-2), TNF-α and IL-1β), antioxidant activity (decreased level of oxidative stress marker—8-oxoguanine) and antiapoptotic activity (decreased B-cell lymphoma 2-associated X protein/B-cell lymphoma protein-2 (BAX/Bcl-2) ratio and reduced caspase-3 and caspase-9 expression) in rats with accelerated aging induced by *d*-galactose administration. Caffeine treatment alleviated synaptic dysfunction (increasing the level of presynaptic proteins synaptophysin and post-synaptic protein PSD95 in the hippocampus) in *d*-galactose-treated rats [119].

The STZ-induced AD model is associated with impaired brain glucose metabolism, Aβ accumulation and tau hyperphosphorylation. STZ *i.c.v.* administration increase oxidative stress, AChE activity and neuroinflammation in the hippocampus and cortex [35,96,116,144]. Chronic caffeine administration prevented STZ-induced progressive memory loss, sporadic dementia and neurodegeneration. It also decreased the expression and density of A_2A_R in the hippocampus (not affecting A_1_R density) in rats [141]. Moreover, caffeine administered to rodents with diabetes mellitus induced by *i.p.* STZ injections prevented learning and memory deficits [145,146].

In the AlCl_3_-induced AD model, administration of aluminum chloride increases level of Aβ, BACE-1, hyperphosphorylated tau protein, proinflammatory cytokines (TNF-α, IL-6) and oxidative stress markers in the hippocampus and cortex [35,147]. Chronic caffeine intake exerted neuroprotective activity demonstrated by improvement of the histological hippocampus picture (decreased neuronal apoptosis observed in CA1 and CA3 hippocampal regions) in AlCl_3_ treated rats. Moreover, increased proliferation marker protein—Ki-67 immunoreactivity, decreased glial fibrillary acidic protein (GFAP) immunoreactivity, and increased expression of BDNF and TrkB were observed after caffeine intake [142]. Further studies on caffeine showed that neuroprotective activity may have resulted from reduced oxidative stress (reduced levels of NO in the cerebral cortex, hippocampus and striatum and increased level of reduced glutathione—GSH in the hippocampus) and attenuated AlCl_3_-induced lipid peroxidation (reduced level of malondialdehyde—MDA in the cerebral cortex, hippocampus and striatum). Caffeine decreased activity of Na^+^/K^+^-ATPase in the cerebral cortex, hippocampus and striatum, and reduced activity of AChE in the cerebral cortex and hippocampus. Also anti-inflammatory properties of caffeine were demonstrated (decreased TNF-α level in the hippocampus and striatum) in AlCl_3_-induced neurotoxicity in rats [143].

LPS administration induces chronic neuroinflammation and amyloidosis leading to cognitive deficits and memory impairment, reflecting processes specific for sporadic type of AD [124]. Increased BACE-1 and γ-secretase activity leading to Aβ hippocampal accumulation, elevated proinflammatory cytokines level (IL-1β, IL-6, IL-12, TNF-α), mitochondrial dysfunction, and increased ROS production is observed in LPS-induced model of AD in rats [124]. Caffeine exerted protective effect against LPS-induced and age-related neuroinflammation associated with microglia activation in rats [122]. It has been demonstrated that caffeine may decrease neuroinflammation by a reduction in the number of activated microglial cells in the CA3 hippocampus region and regulation of glutamate neurotransmission [122]. Caffeine administration inhibited LPS-induced oxidative stress, neuroinflammation and synaptic dysfunctions (increasing expression of nuclear factor erythroid 2-related factor 2 (Nrf2), hemeoxygenase 1 (HO-1) and Bcl-2, reducing expression of toll-like receptor 4 (TLR-4), phosphorylated nuclear factor-κB (p-NF-κB), phosphorylated c-Jun N-terminal kinase (p-JNK), BAX, caspase-3, TNF-α, COX-2 and NOS-2) in mice [123].

Intraperitoneal administration of scopolamine, a non-selective, competitive muscarinic receptor antagonist, is associated with acute memory and attention deficits through cholinergic system blockade [112,115]. Long-term cholinergic dysfunction results in a reduced number of cholinergic neurons, decreased ACh level in the brain, increased AChE activity and suppressed choline acetyltransferase activity. Moreover, scopolamine increases oxidative stress, apoptosis, mitochondrial dysfunction and neuroinflammation in animal AD models [148,149,150]. Although the scopolamine-induced amnesia/memory impairment as a model of AD was widely used in the past, the relevance of scopolamine in current experimental AD studies is very limited [112,114]. Cholinergic dysfunction observed after acute scopolamine administration is not related to the hallmarks of AD (Aβ or hyperphosphorylated tau protein) and disease progression [112]. The effects of caffeine (or selective adenosine receptors antagonists) were studied only after acute scopolamine administration. In scopolamine-treated mice, caffeine administration prevented short-term and long-term memory deficits assessed in the behavioral tests (novel object recognition task and inhibitory avoidance task), indicating possible beneficial effect in cholinergic-induced memory disruption [115]. Caffeine ameliorated also scopolamine-induced memory impairment in humans [151]. The beneficial effect of caffeine probably resulted from its dual ability to blockade of A_1_R and A_2A_R [115]. Studies conducted in scopolamine-treated rats showed that A_1_R and A_2A_R antagonists prevented scopolamine-induced memory impairment [61,152,153].

#### 5.1.3. Rabbit Cholesterol-Induced Model

A high cholesterol diet-induced rabbit model of AD is characterized by an elevated Aβ level in the brain, increased tau protein hyperphosphorylation and disturbed BBB integrity (associated with a disrupted brain cholesterol homeostasis) leading to the learning impairment [154]. Chronic caffeine administration to rabbits fed a cholesterol-enriched diet prevented dysfunction of BBB, decreased activation of astrocytes and decreased density of microglia [155] (Table 3). In another study, caffeine intake decreased Aβ level, Aβ production and Aβ deposition in the hippocampus, reduced hyperphosphorylated tau protein level in the hippocampus (probably associated with the reduction of phosphorylated glycogen synthase kinase-3β (pGSK-3β) enzyme level which is involved in the tau protein phosphorylation), decreased oxidative stress (reduced ROS generation and H_2_O_2_ production, and increased reduced/oxidized glutathione (GSH/GSSG) ratio) and restored A_1_R level, reduced by cholesterol. There was no caffeine effect on the cholesterol concentration in plasma in rabbits [156].

#### 5.1.4. Nematode Models

Nematodes are suitable for experimental studies on AD due to their short lifespan, short generation time, transparent body and simple creation of new transgenic lines. Many human genes associated with AD have orthologues in nematodes *Caenorhabditis elegans* [112,157]. Various strains of the nematode *Caenorhabditis elegans* with human neurotoxic proteins (Aβ_1-42_, APP, tau protein) expression are used to investigate the cellular and molecular mechanisms of neurodegenerative diseases [112,158,159,160]. Expression of the human Aβ_1-42_ in muscles causes, among other things, development of paralysis, whereas its expression in neurons results in their neurodegeneration, and odor preference learning disruption [96,159]. After expression of the human APP in *Caenorhabditis elegans*, only products of α-secretase or γ-secretase cleavage (but not β-secretase) are detected, and APP-induced cholinergic neurodegeneration is observed [159]. Expression of the h-tau protein in *Caenorhabditis elegans* leads to tau aggregate formation and accumulation, neuronal degeneration and synaptic abnormalities resulting in locomotion defects and behavioral impairment [96,159].

In studies conducted on the nematode AD models, plant extracts containing caffeine were investigated, including the extracts from *Ilex paraguariensis* leaves [161], *Paullinia cupana* (guarana) seeds [158], *Coffea* beans [160], and Zijuan Pu’er tea [162] (Table 4). All studied extracts prevented Aβ-induced toxicity in transgenic models of AD in *Caenorhabditis elegans*, delaying the paralysis progression in worms and extending their lifespan [158,160,161,162]. However, coffee extract (10% *v*/*v*, containing 3.6 mM caffeine) treatment did not reduce Aβ aggregation and Aβ distribution. The authors concluded that beneficial effect of coffee probably resulted from *skn*-1/Nrf2 signaling pathway activation; similar effects were observed after treatment with decaffeinated coffee (0.032 mM caffeine) [160]. A mixture of Zijuan Pu’er tea water extract ingredients (MCCP), containing (+)-catechins, caffeine and procyanidins, decreased Aβ aggregation and activated the DAF-16 signaling pathway, which was associated with lifespan extension and oxidative stress reduction in *Caenorhabditis elegans*, and could be mediated by a heat shock factor 1 (HSF-1) and *skn*-1 [162]. Guarana hydroalcoholic extract and decaffeinated guarana extract treatments were also associated with antioxidant activity and protein degradation pathways, partially through *skn*-1 and DAF-16 activation [158].

Caffeine alone was investigated only in one study on nematodes [161]. The effect of caffeine treatment in higher concentrations (0.2 mM or 0.4 mM) was weaker than the effect of *Ilex paraguariensis* hydroalcoholic extract (IPHE) containing lower concentrations of caffeine (41 or 87 μM). IPHE and caffeine reduced Aβ mRNA levels, decreased AChE activity, increased expression of hsp-16.2 (chaperone protein which overexpression causes suppression of Aβ-toxicity), but also activated DAF-16 signaling (IPHE also HSF-1). It may suggest that beneficial neuroprotective effects may result from different than caffeine constituents of IPHE [161]. Decaffeinated extracts from coffee [160] and guarana [158] also induced some favorable effects in *Caenorhabditis elegans*.

In conclusion, the studies on nematode models of AD did not allow the effects of caffeine to be distinguished from those of other constituents of plant extracts [158,160,161].

### 5.2. In Vitro Studies

Caffeine administered to the culture medium caused an enhanced release of stored Ca^2+^ in the in vitro culture of the cortical neurons of 3xTg-AD mice (Table 5). The effect was not associated with a change in the endoplasmic reticulum store size, defects in the Ca^2+^ extrusion mechanism or expression of Ca^2+^-binding/buffering proteins, but probably with an increased expression of the RyR [107]. Similar results were obtained in later studies showing increased calcium signals within dendritic processes induced by RyRs stimulation by caffeine. The proper regulation of calcium signaling may be important in the prevention of synapse loss and further cognitive impairment [163]. Beneficial effect of caffeine treatment was demonstrated in vitro in cultures of human neuroblastoma SH-SY5Y cells, to which Aβ was added in order to induce neurotoxicity [164,165]. Caffeine increased expression of antiapoptotic protein Bcl-2 and reduced expression of proapoptotic protein BAX in the AlCl_3_-induced and Aβ_25–35_-induced neurotoxicity in human neuroblastoma SH-SY5Y cells. Caffeine inhibited also the increase of AD-related proteins (APP and BACE-1) expression in cells exposed to both AlCl_3_ and Aβ_25–35_. The effects of caffeine were similar to those induced by selective A_1_R or A_2A_R antagonists, indicating the role of blockade of both A_1_R and A_2A_R. Caffeine reduced also oxidative stress (reducing NF-κB activity, reducing ROS production, increasing superoxide dismutase (SOD) activity and decreasing MDA concentration) induced by AlCl_3_ and Aβ_25–35_. The authors proposed that combined involvement of A_1_R and A_2A_R blockade by caffeine in the neuronal cell protection is associated with the restoration of Ca^2+^ homeostasis [164]. In another study, the neuroprotective activity of caffeine against Aβ-induced neurotoxicity was confirmed in a neuronal cell line. The effects were shown to result from complex mechanism of caffeine action—the blockade of A_1_R and A_2A_R, direct or indirect blockade of NMDA receptors and activation of RyRs [165].

Viral delivery of mutated human APP and tau protein to primary rat hippocampal neurons and rat dorsal root ganglion (DRG) caused accelerated neuronal cell death and morphological damage (more severe effects were observed in tau transduced cultures). Also, calcium homeostasis dysregulation was observed in APP-EGFP and tau-DsRed2 transduced hippocampal neurons. Moreover, a neurite impairment was demonstrated in APP-EGFP and tau-DsRed2 transduced DRG neurons. Caffeine treatment prevented morphological neuronal damage increasing the number of healthy neurons in APP-induced and tau-induced models [166].

### 5.3. In Silico Studies

In silico studies indicated that caffeine may be useful as an anti-amyloidogenic agent in the prevention of AD [167]. Caffeine was studied in silico as a potential molecule that destabilizes preformed Aβ protofilaments. The molecular dynamics simulations and calculations indicated that the mechanism of caffeine action may be associated with the disruption of inter-chain hydrogen bonds and disorganization of secondary structure conformation of Aβ [167]. In another study, molecular dynamic simulations demonstrated that caffeine inhibited the self-assembly of Aβ oligomerization by interference with the hydrophobic interaction between Aβ_16-22_ peptides; the effect was greater when caffeine concentration in the water solution was higher [168]. In silico studies predicted caffeine to be a potential AChE inhibitor [169]. An improvement of cholinergic system transmission was also revealed in silico molecular docking study, where a coffee and green tea constituent—EGCG inhibited AChE and butyrylcholinesterase [170].

## 6. Discussion

So far no drugs have been proven to be effective in reversing or stopping AD. Despite numerous studies having been conducted in recent years, no new drugs have been registered by the FDA for almost 20 years [171]. Due to the not fully understood, complex pathomechanism of AD and numerous clinical failures of anti-amyloid and anti-tau drugs, it is necessary to look at strategies involving modifying factors connected with the style of life or dietary factors which potentially would counteract the development of neurodegenerative changes or delay AD progression. Caffeine, or rather its sources (coffee, tea, yerba mate), are considered factors which may exert beneficial effects in the AD—prevent the risk of developing AD and/or delay the progression of AD [81,82].

To investigate the effects of caffeine in AD, numerous studies have been undertaken on different experimental models. In those studies, caffeine was administered in different doses. Caffeine was administered in drinking water, in the diet, or once/twice daily *p.o.* or *i.p*. The descriptions of the doses (low, moderate, high) used by authors in their reports on in vitro and in vivo studies are not unequivocal. Caffeine consumption in humans differs in a wide range. A low caffeine intake is considered to be below 200 mg/day (<2.86 mg/kg/day), moderate between 200 and 400 mg/day (2.86–5.71 mg/kg/day) and high above 400 mg/day (>5.71 mg/kg/day) [8,172]. Based on the body surface area conversion ratio, the doses administered to humans are converted into animal doses. Thus, based on the calculations [173], the corresponding rat doses are as follows: low (<17.7 mg/kg/day), moderate (17.7–35.4 mg/kg/day) and high (>35.4 mg/kg/day). Equivalent mouse doses under 35.1 mg/kg/day are considered as low, between 35.1 and 70.3 mg/kg/day as moderate and above 70.3 mg/kg/day as high [172,173,174]. In most of the issues discussed in the present article about animal studies, caffeine was administered in doses corresponding to the human doses; the exact doses used in particular experiments are shown in Table 1, Table 2 and Table 3.

Generally, experimental studies on caffeine effects in AD demonstrated some beneficial influence on cognition. Its neuroprotective, antioxidant, anti-inflammatory and antiapoptotic activities in the neuronal tissues, led to alleviation of cognitive impairment [104,119,122,123,128,131,132,133,136,141,142,143,155,156,158,160,161,164,165,166]. Many studies pointed out on favorable effects of caffeine on the hallmarks of the disease: Aβ or hyperphosphorylated tau protein [104,117,129,131,134,156,161,162,164,165,166].

Since the main mechanism of caffeine action is associated with a non-selective blockade of adenosine receptors (mainly A_1_R and A_2A_R), this problem was undertaken in many studies. In fact, both adenosine receptors (A_1_R and A_2A_R) are present in synapses of glutamatergic, GABAergic, cholinergic, dopaminergic, serotoninergic and noradrenergic system, and adenosine is involved in neuromodulation of the central nervous system (CNS) [175]. Adenosine has been shown to reduce the release of various neurotransmitters including glutamate, ACh, dopamine, serotonin and noradrenaline in CNS in experimental models, whereas caffeine, as an adenosine receptor antagonist, to promote the release [22].

In fact, the favorable effects of caffeine were shown to result from the blockade of A_2A_R (but probably not A_1_R) in mice with AD induced by Aβ_25–35_ *i.c.v.* administration [117], in Aβ-induced neurotoxicity in the primary cerebral cultures in rats [176] and in STZ-induced AD in rats [141]. However, A_1_R may be involved in the protective caffeine activity in cholesterol-enriched diet-induced AD in rabbits [156]. Moreover, the blockade of both A_1_R and A_2A_R was responsible for beneficial activity of caffeine in in vitro model of combined neurotoxicity induced by AlCl_3_ and Aβ_25–35_ in human neuroblastoma SH-SY5Y cells [164] and in scopolamine-induced memory deficit in mice [115]. Dual blockade was also considered a probable mechanism of caffeine neuroprotective activity in Aβ-induced toxicity in human neuroblastoma SH-SY5Y cells [165].

Beneficial effects of caffeine resulting from the blockade of adenosine receptors (A_1_R and A_2A_R) have been confirmed by studies with selective A_1_R and A_2A_R antagonists. The studies with use of selective A_2A_R antagonists (MSX-3 and SCH58261) demonstrated that A_2A_R blockade prevented spatial memory deficits and development of amyloid burden in the double transgenic APPswe/PS1ΔE9 mice [177] and improved synaptic plasticity deficits in double transgenic APPswe/PS1 mice [178]. Similar favorable effects of selective A_2A_R antagonist administration (SCH58261) were observed also in 3xTg (APPswe, PS1/M146V and tau P301L) mice [179] and in Aβ-induced neurotoxicity and synaptotoxicity in rats [120,140]. Deletion of A_2A_R prevented memory impairment and reduced tau protein hyperphosphorylation in THY-Tau22 transgenic mice [139]. Similar effects were observed after caffeine treatment in THY-Tau22 mice [104]. However, SCH58261 did not favorably affect scopolamine-induced and MK-801-induced acute memory impairment suggesting that selective A_2A_R antagonist exerts beneficial effects only in slowly progressing memory impairment related to synaptic deterioration [120]. However, blockade of A_2A_R (by a selective antagonist—SCH58261), and also A_1_R (by a selective antagonist—DPCPX) led to beneficial effects in a scopolamine-induced model in mice [153], confirming the effect observed after caffeine treatment in this mice model [115], but inconsistent with conclusions drawn by other authors [180].

It should be pointed out that the effects of caffeine on adenosine transmission were not consistent in all studied models. For example, no influence of caffeine on A_1_R and A_2A_R density was observed in APPswe mice [129]. Moreover, chronic caffeine intake did not result in persistent up-regulation of A_1A_R in rat brains excluding this mechanism of action as the reason for the neuroprotective effect of caffeine in rats [181]. It should be emphasized that the mechanism of caffeine action on adenosine system is dose-dependent, since, for example, in mice, acute caffeine intake at lower doses (<50 mg/kg) blocks mostly A_1_R and at higher non-toxic doses (<100 mg/kg) A_2A_R [117].

Although numerous studies indicate that antagonistic effect of caffeine on adenosine receptors may be responsible for alleviation of memory deficits, it is not the only possible mechanism of beneficial caffeine action in AD. From among other caffeine mechanisms, the inhibitory effect on PDEs and agonistic effect on RyRs were investigated in in vitro models of AD disorders. Caffeine inhibited PDE, restoring the mitochondrial functions in vitro in mouse N2a-APPswe cells [133]. Activation of RyRs by caffeine led to an increased Ca^2+^ release from intracellular stores in the cortical cultures from 3xTg-AD mice [107] and in the hippocampal cultures from normal rats [24]. However, the caffeine doses required to inhibit PDEs and stimulate the RyRs are not achievable in a normal diet and could exert toxic effects in humans [9,23,25,26].

Numerous mechanisms of caffeine action, which may be relevant to its favorable effects in AD models were demonstrated in studies on cell cultures. For example, in vitro caffeine decreased Aβ level and Aβ_1-42_ deposition, and reduced BACE-1 activity in human neuroblastoma SH-SY5Y cells [182], and inhibited oligomerization of Aβ in N2a/APP cells [183]. Also decreased expression of BACE-1 and APP was demonstrated after caffeine treatment in human neuroblastoma SH-SY5Y cells exposed to AlCl_3_ and Aβ [164]. In addition, the beneficial effect of caffeine on Aβ could be associated with antagonism of NMDA receptors or agonism of RyRs [165]. Moreover, brewed coffee reduced Aβ production by a decrease in BACE-1 expression (which was not associated with caffeine activity) in human neuroblastoma SH-SY5Y cells [184]. Brewed coffee increased expression of vascular endothelial growth factor (VEGF)in SH-SY5Y cells, indicating possible neuroprotective effect [185]. The effects of brewed coffee treatment suggest that the neuroprotective activity may result not only from caffeine activity, but also from that of other coffee components.

Since AChE inhibitors (donepezil, rivastigmine, galantamine) currently play the main role in the treatment of AD, the effects of caffeine on AChE were also studied. In vivo studies demonstrated weak anticholinesterase activity of caffeine in normal rats [172], in rats with AlCl_3_-induced neurotoxicity [143] and in a nematode AD model [161]. In vitro studies on caffeine and donepezil effects in the rat brain tissue homogenate demonstrated that both drugs inhibited AChE, whereas the effect was stronger after the combined treatment (with stronger inhibition at higher caffeine concentration). On the other hand, in vivo studies did not confirm the augmentation of donepezil anticholinesterase activity by caffeine, moreover administration of high-dose caffeine with donepezil elevated the AChE activity [172]. This may suggest adverse effects of high caffeine consumption during the treatment of AD with AChE inhibitors.

The mechanism of favorable actions of caffeine on AD may be connected with the reduction of neuroinflammation [5,186]. AD and other neurodegenerative diseases are associated with an increased level of inflammation and oxidative stress parameters. Numerous studies showed that caffeine reduced parameters of oxidative stress. Caffeine exerted antioxidant activity in transgenic [104,131] and non-transgenic rodent models [119,123,143], a rabbit model [156], and nematode models [158,161] of AD.

Neuroprotective, anti-inflammatory and antiapoptotic activity of caffeine demonstrated in different experimental models of AD in vivo was reflected in in vitro studies. Caffeine exerted antiapoptotic activity in APPswe transgenic mice [128,131] and in the *d*-galactose-treated rats [119]. Anti-inflammatory effect of caffeine administration was associated with the reduced level of proinflammatory cytokines in THY-Tau22 transgenic mice [104], in AlCl_3_-induced neurotoxicity in rats [143] and in the *d*-galactose-treated rats [119].

**Figure 1:**
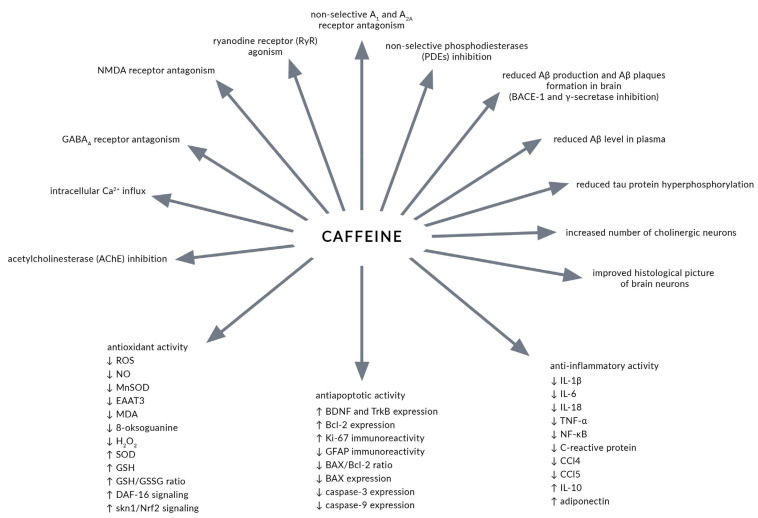
Caffeine effects reported in studies performed in experimental models of Alzheimer’s disease (AD). Aβ—amyloid β. AChE—acetylcholinesterase. BACE-1—β-site amyloid-precursor-protein-cleaving enzyme-1. BAX—B-cell lymphoma 2-associated X protein. Bcl-2—B-cell lymphoma protein-2. BDNF—brain-derived neurotrophic factor. CCl—chemokine ligand factor. DAF-16—abnormal dauer formation 16. EAAT3—excitatory amino acid transporter 3. GABA_A_—γ-aminobutyric acid type A receptor. GFAP—glial fibrillary acidic protein. GSH—reduced glutathione. GSH/GSSG ratio—reduced/oxidized glutathione ratio. IL—interleukin. MDA—malondialdehyde. MnSOD—manganese superoxide dismutase. NF-κB—nuclear factor-κB. NMDA—N-methyl-D-aspartate. Nrf2—nuclear factor erythroid 2-related factor 2. PDE—phosphodiesterase. ROS—reactive oxygen species. RyR—ryanodine receptor. skn-1—skinhead 1. SOD—superoxide dismutase. TNF-α—tumor necrosis factor α. TrkB—tropomyosin-related kinase receptor B.

The caffeine effects presented in studies performed in experimental models of AD are presented in Figure 1.

It must be stated that caffeine shares antioxidant and anti-inflammatory effects, leading to neuroprotection, with numerous phenolic compounds, many of them occurring together with caffeine in its dietary sources (coffee, tea, yerba mate). In fact, crude caffeine (containing 1% of phenolic acids) exerted stronger favorable effects than pure caffeine [131]. Also, other substances present in coffee or tea like chlorogenic acid [187,188,189,190], caffeic acid [79,189,191], EGCG [3,79], ferulic acid [76] exerted beneficial effects in experimental models of AD.

It should be pointed out that caffeine did not exert beneficial effects in all experiments on AD experimental models. Caffeine administration did not favorably affect behavioral disorders studied in the behavioral tests in 3xTg-AD mice. Intensification of BPSD-like behaviors and anxiety-related behaviors was demonstrated after caffeine treatment in those mice [135]. The adverse effect of caffeine intake during pregnancy in THY-Tau22 transgenic rats was demonstrated in the behavioral tests in offspring, in which caffeine accelerated the occurrence of cognitive deficits [105]. Detrimental effects of caffeine intake rather than that of other coffee components during pregnancy (low birth weight of infant, increased risk of pregnancy loss and childhood acute leukemia) was also confirmed in an umbrella review on the impact of coffee and caffeine on health outcomes [14,16].

As was mentioned before, caffeine (as a component of coffee, tea, yerba mate, cola) is the most widely used psychostimulant [8,23,192,193]. The safety of caffeine consumption depends on the dose, age, sex and health condition [12,13,194]. Caffeine intake in coffee, tea, yerba mate, cola and other sources at doses up to 400 mg/day is considered safe for healthy adults, except pregnant women (<300 mg/day) [14,194]. In children and adolescents, caffeine consumption up to 2.5 mg/kg/day was not associated with overt adverse effects [14]. Caffeine intake may negatively affect pregnant women, lactating women, children, adolescents, people with cardiovascular disorders, people with gastric and duodenal ulcer disease and smokers [194,195]. Caffeine intake before bedtime is associated with negative impact on sleep condition, although sleep avoidance and increased vigilance and arousal may be desirable [14]. The adverse caffeine effects on the behavior observed at higher doses intake are anxiety, jitteriness and mood disorders [8,13,14,196]. Negative effect of caffeine consumption was demonstrated also in children, including reduced sleep time, impulsiveness, greater emotional lability and depression [197].

Experimental studies, reviewed in the present article, indicate rather favorable effects of caffeine in animal models of AD. However, results of such studies may not be fully relevant to AD in humans. The AD models have been used to investigate many potential drugs that have proven to be effective in the animals, but this has not necessarily been confirmed in the human studies. Multiple potential disease-modifying drug candidates (γ-secretase inhibitors, BACE-1 inhibitors, monoclonal antibodies, anti-Aβ antibodies, tau aggregation inhibitors) failed in the further phases of the trials [36,51,52,171,198,199].

The studies on transgenic models were limited to the mouse models. The rat studies, carried out on non-transgenic models, were less numerous. In many of the experiments caffeine was administered in drinking water or in the diet, the effect of caffeine used in bolus doses at regular time intervals, over a certain period of time may be different.

Another limitation of the experimental studies is that most of them were carried out on male animals (or the animal sex was not stated), whereas human studies indicate that the effect of caffeine on cognition is sex-dependent [95,200,201]. For example, caffeine intake was associated with better cognitive performance in older women, but not in men [202]. On the other hand, the beneficial effects of caffeine consumption were observed in older men but not in women [200]. Moreover, contradictory conclusions have been drawn from the analysis of the relationship between the sex-dependent effects of caffeine consumption on different cognitive outcomes: there was no significant association between caffeine administration and cognitive performance in men; significant interaction between caffeine intake and cognitive performance was observed only in women [95,201]. The sex-dependent effect of caffeine could result from changes in circulating steroid hormones or differences in the caffeine metabolism (probably resulting from higher xanthine oxidase activity in women) in female and male organisms [95,200].

## 7. Conclusions

In conclusion, the studies carried out on experimental models generally support the notion that dietary caffeine consumption may exert some beneficial effects in AD. However, further studies are necessary to elucidate the role of caffeine in the effects of its sources on cognition and possibly AD risk.

## Figures and Tables

**Table 1:** Transgenic models.

Model	Caffeine Dose and Study Conditions	Aim	Effects	Reference
APPswe (K670N/ M671L) male and female mice	Caffeine: 0.3 g/L *p.o.* in drinking water for 5.5 months; start: 4-month-old mice, 8-month-old at the start of behavioral tests, 9.5-month-old at the end of behavioral tests	To investigate the possible neuroprotective effects of long-term dietary caffeine intake in APPswe transgenic mice.	Caffeine protected against cognitive impairment. It reduced Aβ levels in the hippocampus, restored brain adenosine levels, but did not affect the A_1_R and A_2A_R hippocampal density and expression in the cerebral cortex and hippocampus.	[129]
APPswe (K670N/ M671L) mice	Caffeine: 1.5 mg *p.o. * for 2 weeks, every 12 h; start: 9.5-month-old mice	To investigate the effects of caffeine on the signal transduction pathways (PKA, CREB, JNK and ERK) in cognitively important areas of the brain.	Caffeine showed beneficial effects in the brain function and exerted neuroprotective and antiapoptotic effect by stimulating PKA activity, increasing the level of phosphorylated CREB and decreasing JNK and ERK phosphorylation.	[128]
APPswe (K670N/ M671L) mice	Caffeine: 0.6 mg/day in drinking water for 1 month; start: 11- to 12-month-old mice	To explain the protective mechanism of caffeine and melatonin administration against cognitive dysfunction in transgenic APPswe mice.	Caffeine and melatonin prevented cognitive impairment. Caffeine slightly increased mitochondrial functions, however it inhibited the enhancement of mitochondrial functions provided by melatonin.	[133]
APPswe (K670N/ M671L) and Indiana (V717F) mutation male mice	0.0395% crude caffeine (95.95% caffeine, 1.1% moisture, 1.04% fat, 0.1% ash) in diet or 0.0375% pure caffeine in diet for 2 months; start: 3-month-old mice	To investigate the effects of consumption of crude and pure caffeine on the learning and memory processes in transgenic AD mice.	Crude and pure caffeine administered for two months partly prevented memory deficits (crude caffeine exerted greater effect). Crude caffeine (but not pure) reduced Aβ_1-42_ levels, suppressed Aβ accumulation and reduced the number of Aβ plaques in the hippocampus. Both prevented Aβ-induced neuronal cell death and exerted antiapoptotic activity suppressing caspase-3 activity. Antioxidant and anti-inflammatory effects of crude caffeine were also demonstrated in APPswe mice.	[131]
APPswe mice and APPswe/PS1 mice	Caffeine: 1.5 mg *i.p.*, single administration, 3- to 4-month-old APPswe mice; caffeine: 1.5 mg *i.p.* or *p.o.*, single administration, 14-month-old APPswe mice; caffeine: 1.5 mg *i.p.,* single administration, 14-month-old APPswe/PS1 mice; caffeine: 1.5 mg *p.o.* twice-daily for 7 days, 15- to 20-month-old APPswe/PS1 mice; caffeine: 1.5 mg *p.o.*, two administrations on one day every 4t^h^ day for 2 months, 20-month-old APPswe/PS1 mice;	To investigate the effects of acute and long-term caffeine administration on the cognitive performance and Aβ levels in APPswe and APPswe/PS1 transgenic mice.	Long-term caffeine intake improved cognitive functions in APPswe and APPswe/PS1 mice. Decreased Aβ levels in the plasma were observed after single administration of caffeine and long-term caffeine treatments in both transgenic mice models. Chronic caffeine treatment reduced soluble Aβ level in the cortex and hippocampus and insoluble Aβ level in the hippocampus in APPswe mice. Acute caffeine administration rapidly reduced the Aβ level also in the interstitial fluid in the hippocampus but did not affect Aβ elimination in APPswe mice.	[134]
APPswe/PS1 mice and APPswe mice, control non-transgenic mice	Caffeine: 0.3 mg (unconcentrated coffee) *i.p.*, single administration or 1.5 mg (concentrated coffee) *i.p.*, single administration or 0.06 mg (concentrated decaffeinated coffee) *i.p.*, single administration, 6- to 8-month-old APPswe/PS1 transgenic mice; caffeine: 0.75 mg (concentrated coffee) *p.o.*, twice weekly for 3 months or 0.03 mg (decaffeinated coffee) *p.o.*, twice weekly for 3 months, 10-month-old APPswe transgenic mice	To investigate the effects of acute and long-term treatment with coffee or decaffeinated coffee (to compare the effect of caffeine to coffee) on the plasma cytokine and Aβ levels and behavior (only after long-term treatment) in transgenic mice models of AD.	Acute caffeine intake increased the level of G-CSF and IL-10 in plasma (concentrated and unconcentrated coffee) and IL-6 level in plasma (only concentrated coffee). Higher plasma caffeine concentrations were related to lower levels of Aβ in plasma. Single treatment with coffee (concentrated and unconcentrated) increased G-CSF, IL-6 and IL-10 plasma levels also in non-transgenic mice. Long-term treatment with concentrated coffee (but not decaffeinated coffee) favorably affected cognitive interference task and elevated level of G-CSF (but not other cytokines) in plasma in transgenic mice. Improvement of cognitive performance was associated with higher G-CSF levels suggesting that elevated G-CSF levels may be associated with possible beneficial effects of coffee against AD.	[136]
APPswe/PS1 mice	Caffeine: 0.75 mg/day or 1.5 mg/day *p.o.* for 8 weeks; 12-month-old mice	To investigate the effects of caffeine intake on the memory deficits, BDNF and TrkB expression in APPswe/PS1 double transgenic mice.	Caffeine at both doses used increased spatial learning ability and memory capability. Caffeine treatment increased the expression of hippocampal BDNF and TrkB. Caffeine exerted protective role against memory impairment in APPswe/PS1 mice.	[132]
THY-Tau22 male mice	Caffeine: 0.3 g/L *p.o.* in drinking water for 10 months; start: 2-month-old mice	To investigate the effects of chronic caffeine intake on the development of hippocampal tau protein pathologies and spatial memory disorders in THY-Tau22 transgenic mice.	Chronic caffeine intake prevented spatial memory deficits and improved memory performance. Caffeine effect was associated with a reduction of neuroinflammation and decrease in the hippocampal level of hyperphosphorylated tau protein. Caffeine treatment decreased oxidative stress (reduced expression of MnSOD and EAAT3) in THY-Tau22 mice.	[104]
THY-Tau22 female mice	Caffeine: 0.3 g/L *p.o.* in drinking water; start of caffeine administration: 2 weeks before mating; end of caffeine administration: 15^th^ postnatal day; 8- or 12-month-old at the start of behavioral tests.	To investigate the effects of long-term caffeine exposure during pregnancy in offspring in THY-Tau22 transgenic mice.	The exposure to caffeine during pregnancy induced physiological disorders and accelerated cognitive disorders in THY-Tau22 transgenic mice model and may be a risk factor for early stages of AD.	[105]
3xTg (APPswe, PS1/M146V and tau P301L) male mice, control non-transgenic mice	Caffeine: 0.3 mg/mL in drinking water *p.o.* for 7 months; start: 6-month-old mice; behavioral testing at 13 months of age.	To investigate the effects of long-term caffeine administration on the memory and learning in 3xTg-AD mice with behavioral and psychological symptoms of dementia (BPSD) profile.	Caffeine increased motor activity, total horizontal activity and emotionality in the behavioral tests in non-transgenic mice and reduced it in 3xTg-AD mice. Caffeine administration increased spontaneous motor activity (to a greater extent at night) only in 3xTg-AD mice. Results indicate that aggravation of BPSD-like behaviors, anxiety-related behaviors or neophobia adversely affected possible beneficial effects of caffeine treatment (improvement of memory and learning) in 3xTg-AD mice.	[135]

3xTg—triple-transgenic. A_1_R—adenosine A_1_ receptor. A_2A_R—adenosine A_2A_ receptor. Aβ—amyloid β. AD—Alzheimer’s disease. BDNF—brain-derived neurotrophic factor. BPSD—behavioral and psychological symptoms of dementia. CREB—cAMP response element-binding protein. EAAT3—excitatory amino acid transporter 3. ERK—extracellular signal-regulated kinase. G-CSF—granulocyte-colony stimulating factor. IL—interleukin. *i.p.*—intraperitoneally. JNK—c-Jun N-terminal kinase. MnSOD—manganese superoxide dismutase. Nrf2—nuclear factor erythroid 2-related factor 2. PKA—phosphokinase A. *p*.*o*.—per os. TrkB—tropomyosin-related kinase receptor B.

**Table 2:** Non-transgenic models.

Model	Caffeine Dose and Study Conditions	Aim	Effects	Reference
Adult CF1 male mice administered with Aβ_25–35_ *i.c.v.* (3 nmol; volume: 3 μL)	Caffeine: 1 mg/mL *p.o.* in drinking water (22 mg/kg/day) for 12 days, Aβ *i.c.v.* on 7th day; caffeine: 30 or 80 mg/kg *i.p.*, single administration, 30 min before Aβ *i.c.v. * caffeine: 30 mg/kg for 4 days, Aβ *i.c.v*. after 2 days of caffeine intake; caffeine: 1 mg/mL *p.o. * in drinking water (for 12 days) and 30 mg/kg *i.p.*, single administration, 30 min before Aβ *i.c.v*.; behavioral tests were performed 8–9 days after Aβ *i.c.v.* administration.	To investigate the effects of caffeine and a selective A_2A_R antagonist against cognitive impairment in AD induced by *i.c.v.* Aβ_25–35_ administration in mice.	Blockade of A_2A_R by caffeine or by a selective A_2A_R antagonist prevented cognitive impairment, neurodegeneration and brain destruction in Aβ-induced AD mice model.	[117]
Adult male Sprague-Dawley rats with accelerated aging induced by *d*-galactose administration (120 mg/kg *i.p.* for 60 days)	Caffeine: 3 mg/kg/day *i.p.* for 60 days (during *d*-galactose treatment period)	To investigate the effects of chronic caffeine intake on neurodegeneration induced by *d-*galactose-aging rat model.	Chronic caffeine intake reduced oxidative stress (decreased 8-oxoguanine level), neuroinflammation, neuronal cells apoptosis, neurodegeneration, synaptic dysfunction and memory deficits induced by *d-*galactose injections.	[119]
Adult male Wistar rats with AD induced by *i.c.v.* administration of STZ (3 mg/kg; single bilateral administration)	Caffeine: 1 g/L in drinking water for 2 weeks before and 4 weeks after STZ administration. Behavioral tests 4 weeks after STZ administration	To investigate the effects of caffeine intake on the expression and density of adenosine receptors and hippocampal neurodegeneration in STZ-induced rat model of AD.	Caffeine administration prevented the STZ-induced memory deficits, sporadic dementia, neurodegeneration and decreased expression and density of A_2A_R in the hippocampus.	[141]
Adult male Sprague-Dawley rats treated with AlCl_3_ (17 mg/kg *p.o.* for 4 weeks)	Caffeine 1.5 mg/kg *p.o. * for 4 weeks concurrently with AlCl_3_ or two weeks before the start and for 4 weeks during AlCl_3_ administration	To investigate the effects of caffeine on the histological picture of hippocampus, expression of BDNF, TrkB, and immunoreactivity of Ki-67 and GFAP in the hippocampus in rats with AlCl_3_-induced AD.	Caffeine intake exerted neuroprotective activity (improvement of histological hippocampus picture, stronger Ki-67 and weaker GFAP immunoreactivity, increased BDNF and TrkB gene expression). The effects were stronger in rats treated with caffeine also before the start of AlCl_3_ administration.	[142]
Adult male Wistar rats treated with AlCl_3_ (100 mg/kg *p.o.* for 30 days)	Caffeine: 20 mg/kg *i.p.* for 30 days, 1 h before AlCl_3_ *p.o.* intake	To evaluate an antioxidant, anti-inflammatory and anticholinesterase properties of caffeine against AlCl_3_-induced neurotoxicity in rats.	Caffeine exerted neuroprotective, antioxidant and anticholinesterase activity against AlCl_3_-induced neurotoxicity in rats. It reduced oxidative stress parameters (NO level), decreased AChE and Na^+^/K^+^-ATPase activity in the cerebral cortex and hippocampus (Na^+^/K^+^-ATPase activity also in the striatum). Caffeine revealed also anti-inflammatory properties by reducing the increased TNF-α levels in the hippocampus and striatum associated with AlCl_3_-induced neurotoxicity.	[143]
Fisher-344 young male rats (3-month-old) treated with LPS (0.250 µg/h *i.c.v. * by osmotic minipump for 4 weeks); Fisher-344 male aged rats (24-month-old)	Caffeine: 0.5, 5, 10, 20 or 40 mg/kg/day *i.p.* for 2 or 4 weeks to young rats; caffeine: 40 mg/kg/day *i.p.* for 2 weeks to aged rats	To investigate the effects of different caffeine doses in LPS-induced neuroinflammation in young rats and in age-related neuroinflammation in aged rats (with naturally increased level of microglia activation).	Caffeine exerted potential protective effect against LPS-induced neuroinflammation. It was demonstrated that caffeine may decrease neuroinflammation by a reduction in the number of activated microglial cells in the hippocampus and through regulation of glutamate neurotransmission.	[122]
C57BL/6N male mice treated with LPS (250 µg/kg *i.p.* for 2 weeks; 7 doses in 2 weeks)	Caffeine: 30 mg/kg/day *i.p.* for 6 weeks	To investigate the effect of caffeine administration against LPS-induced oxidative stress, neuroinflammation, apoptotic cell death, neurodegeneration and synaptic impairment in mice.	Caffeine reduced LPS-induced oxidative stress, neuroinflammation and synaptic dysfunctions (increased expression of Nrf2, HO-1 and Bcl-2, reduced expression of TLR-4, p-NF-κB, p-JNK, BAX, caspase-3, TNF-α, COX-2, NOS-2 and synaptic markers) in mouse brains.	[123]
CF1 male mice (3-4-month-old) with cholinergic blockade induced by a single scopolamine hydrobromide administration (2 mg/kg *i.p.*)	Caffeine: 10 mg/kg *i.p*. for 4 days before scopolamine hydrobromide administration; scopolamine administration: 15 min before or immediately after the inhibitory avoidance test, immediately after training session in the novel object recognition task, 90 min before the open field test; independent group of mice for each test	To investigate the effect of caffeine intake on short-term and long-term memory impairment induced by a single scopolamine administration, assessed in three behavioral tests.	Pretreatment with caffeine prevented scopolamine-induced impairment in the acquisition phase when short-term memory was assessed in the inhibitory avoidance task, and in the consolidation phase when short-term and long-term memory were assessed in the inhibitory avoidance task. Caffeine administration prevented scopolamine-induced short-term and long-term memory deficits (assessed in the novel object recognition task). Caffeine exerted beneficial effect in the cholinergic-induced memory disruption. There was no effect of caffeine treatment on the spontaneous locomotor activity assessed in the open field test.	[115]

A_2A_R—adenosine A_2A_ receptor. Aβ—amyloid β. AChE—acetylcholinesterase. AD—Alzheimer’s disease. AlCl_3_—aluminum chloride. ATP—adenosine triphosphate. BAX—B-cell lymphoma 2-associated X protein. Bcl-2—B-cell lymphoma protein-2. BDNF—brain-derived neurotrophic factor. COX-2—cyclooxygenase-2. GFAP—glial fibrillary acidic protein. HO-1—hemeoxygenase 1. *i.c.v.*—intracerebroventricularly. *i.p.*—intraperitoneally. LPS—lipopolysaccharide. NOS-2—nitric oxide synthase-2. Nrf2—nuclear factor erythroid 2-related factor 2. p-JNK—phosphorylated c-Jun N-terminal kinase. p-NF-κB—phosphorylated nuclear factor-κB. *p.o.*—per os. STZ—streptozotocin. TNF-α—tumor necrosis factor α. TLR-4—toll-like receptor 4. TrkB—tropomyosin-related kinase receptor B.

**Table 3:** Rabbit model.

Model	Caffeine Dose and Study Conditions	Aim	Effects	Reference
New Zealand white rabbits (1.5- to 2-years-old) fed a 2% cholesterol-enriched diet	Caffeine: 3 mg/day in 50 mL of drinking water for 12 weeks	To investigate the effects of caffeine on BBB leakage in rabbits fed a cholesterol-enriched diet as a model of sporadic AD.	Chronic caffeine administration prevented dysfunction of BBB, decreased activation of astrocytes and decreased density of microglia induced by high cholesterol diet in rabbits.	[155]
New Zealand white male rabbits (1.5- to 2-years-old) fed a 2% cholesterol-enriched diet	Caffeine: 0.5 mg/day or 30 mg/day in drinking water for 12 weeks	To investigate the effects of caffeine treatment on molecular mechanisms of AD-like pathology in the cholesterol-fed rabbit model of AD.	Chronic caffeine intake decreased Aβ accumulation in the hippocampus and reduced hyperphosphorylated tau protein level in the hippocampus (reduced phosphorylated GSK-3β enzyme level). Caffeine prevented oxidative damage (reduced ROS generation and H_2_O_2_ production, increased GSH/GSSG ratio) and restored the level of A_1_R, reduced by cholesterol in rabbits. Caffeine did not affect the level of A_2A_R and RyRs and the cholesterol concentration in plasma.	[156]

A_1_R—adenosine A_1_ receptor. Aβ—amyloid β. AD—Alzheimer’s disease. BBB—blood-brain barrier. GSH/GSSG—reduced/oxidized glutathione. GSK-3β—glycogen synthase kinase-3β. ROS—reactive oxygen species. RyR—ryanodine receptor.

**Table 4:** Nematode models.

Model	Caffeine Dose and Study Conditions	Aim	Effects	Reference
*Caenorhabditis* *elegans* strains: wild-type N2, CL2006 dvIs2	*Ilex paraguariensis* hydroalcoholic extract (IPHE): 2 or 4 mg/mL (41 or 87 μM caffeine); caffeine: 200 or 400 μM. Treatment since first larval stage till required age for test.	To investigate the effects of IPHE and caffeine administration on the Aβ-induced toxicity in *Caenorhabditis elegans*.	IPHE and caffeine extended lifespan of worms, decreased AChE activity and reduced Aβ deposition and toxicity leading to worms’ paralysis. IPHE and caffeine reduced also Aβ mRNA levels and increased expression of hsp-16.2 (chaperone protein which overexpression causes suppression of Aβ-toxicity). After both treatments an antioxidant activity (reduced ROS levels) was observed.	[161]
Various *Caenorhabditis* *elegans* strains	10% coffee extract (3.6 mM caffeine);	To investigate the effects of coffee extract treatment on the Aβ-induced toxicity in *Caenorhabditis elegans*.	Coffee extract prevented Aβ-induced toxicity in the transgenic models of AD in *Caenorhabditis elegans.* It induced also a delay in the paralysis progression in worms. No reduction in Aβ expression, Aβ aggregation and distribution was observed in coffee-treated group. The beneficial effect of coffee may result from *skn*-1/Nrf2 pathway induction.	[160]
*Caenorhabditis* *elegans* strains: wild-type N2, CL4176 dvls27, TJ356 zIs356, CF1553 muIs84	0.1, 0.2 and 0.4 mg/mL Zijuan Pu’er tea water extract (ZTWE) containing: (+)-catechins, caffeine, procyanidins. Mixture of three main constituents in ZTWE: (+)-catechins, caffeine, procyanidins—MCCP.	To investigate the effects of ZTWE and MCPP on the Aβ-induced toxicity in various *Caenorhabditis* *elegans* strains.	ZTWE and MCCP delayed Aβ-induced paralysis in worms. MCCP alleviated AD progression and pathologies related to AD due to reduced Aβ-induced toxicity (decreased Aβ aggregation), and increased antioxidant activity (activated DAF-16 signaling pathway associated with oxidative stress resistance; decreased ROS production).	[162]
*Caenorhabditis elegans* strains: wild-type N2, CL4176 dvls27, CL2006, AM141, HA759, rtIs11, TJ375, CL2166	Guarana hydroalcoholic extract (GHE) containing: caffeine: 166.1 μg/mL, theobromine: 2.5 μg/mL, catechin: 34.6 μg/mL, epicatechin: 36.3 μg/mL. 10 or 50 mg/mL GHE	To investigate the effects of GHE treatment in *Caenorhabditis elegans * models of AD.	GHE prevented Aβ-induced toxicity in the transgenic models of AD in *Caenorhabditis elegans*. GHE delayed paralysis in nematodes, reduced ROS level and activated protein degradation. DAF-16 and *skn*-1 are responsible for the beneficial effect against Aβ-induced toxicity.	[158]

Aβ—amyloid β. AChE—acetylcholinesterase. AD—Alzheimer’s disease. BBB—blood-brain barrier. DAF-16—abnormal dauer formation 16. GHE—guarana hydroalcoholic extract. GSH/GSSG—reduced/oxidized glutathione. hsp—heat shock protein. IPHE—*Ilex paraguariensis* hydroalcoholic extract. MCPP—mixture of three main constituents in Zijuan Pu’er tea water extract. Nrf2—nuclear factor erythroid 2-related pGSK-3β—phosphorylated glycogen synthase kinase-3β. ROS—reactive oxygen species. *skn-1*—skinhead 1. ZTWE—Zijuan Pu’er tea water extract.

**Table 5:** In vitro models.

Model	Caffeine Dose and Study Conditions	Aim	Effects	Reference
Primary cortical neurons from 12- to 17-day-old cultures from 3xTg (APPswe, PS1/M146V KI and tau P301L) mouse embryos	Caffeine: 25 mM	To investigate the effects of caffeine treatment on disorders in Ca^2+^ homeostasis in the primary cortical neurons obtained from 3xTg-AD mice, assessed by microfluorimetric measurements of Ca^2+^ concentration.	Caffeine treatment increased Ca^2+^ content in the cortical neurons of 3xTg-AD mice. Caffeine increased release of Ca^2+^ from RyR-sensitive Ca^2+^ stores. Enhanced Ca^2+^ response to the caffeine was probably associated with an increased expression of RyRs in the cortical neurons.	[107]
Prefrontal cortex brain slices from 3xTg (APPswe, PS1/M146V KI, and tau P130L) mice (1- to 3-month-old)	Caffeine: 10 mM	To investigate the relationship between Ca^2+^ influx by NMDA receptors and RyR activation in 3xTg-AD mice.	Caffeine stimulated RyRs increasing synaptic excitability. RyR and NMDA receptor activation increased Ca^2+^ release in 3xTg-AD mice.	[163]
Human neuroblastoma SH-SY5Y cells treated with 2 μM Aβ_25–35_ or 10 μM AlCl_3_ or combined	Caffeine: 10 μM; caffeine: 1-100 μM in the cell viability test	To investigate the role of A_1_R and A_2A_R in the neuroprotective activity of caffeine in the AlCl_3_- and Aβ_25–35_-induced models of AD in human SH-SY5Y neuroblastoma cells.	Neuroprotective effect of caffeine observed in the combined AlCl_3_- and Aβ_25–35_ -induced model of neurotoxicity required a dual antagonism of A_1_R and A_2A_R (probably due to combined involvement in the restoration of Ca^2+^ homeostasis). Caffeine prevented neuronal cell death and exerted antioxidant activity (reduced ROS production, increased SOD activity and decreased MDA concentration).	[164]
Human neuroblastoma SH-SY5Y cells treated with 20 μM Aβ_25–35_	Caffeine: 0.6 or 1 mM	To investigate the mechanism of neuroprotective activity of caffeine against Aβ-induced neurotoxicity in the human neuroblastoma SH-SY5Y cells.	Caffeine prevented Aβ-induced toxicity in neuronal cells, which probably resulted from the blockade of adenosine receptors (A_1_ and A_2A_), blockade of NMDA receptors and activation of RyRs.	[165]
Primary hippocampal neurons from 2- to 5-day-old Sprague-Dawley rats or dorsal root ganglion (DRG) from 1- to 4-day-old Sprague-Dawley rats; both transfected with human APP fused to EGFP or mutant h-tau protein fused to DsRed2	Caffeine: 50 μM	To investigate the effects of caffeine and various drugs treatment after viral delivery of mutated APP and h-tau protein in the primary hippocampal cells and DRG cells on the degeneration and neuronal cell death.	Experiments demonstrated that delivering mutated APP and h-tau protein accelerated neuronal cell death and morphological damage. Caffeine administration ameliorated APP-induced and tau-induced neuronal damage. Caffeine treatment exerted neuroprotective effects in APP-induced and tau-induced models (prevented morphological damage in both models, increasing the number of healthy neurons).	[166]

3xTg—triple-transgenic. A_1_R—adenosine A_1_ receptor. A_2A_R—adenosine A_2A_ receptor. Aβ—amyloid β. AD—Alzheimer’s disease. AlCl_3_—aluminum chloride. APP—amyloid precursor protein. DRG—dorsal root ganglion. EGFP—enhanced green fluorescent protein. h-tau—human tau. MDA—malondialdehyde. NMDA—N-methyl-D-aspartate. ROS—reactive oxygen species. RyR—ryanodine receptor. SOD—superoxide dismutase.
